# Annual and Analytical Cyclopaedia of Practical Medicine

**Published:** 1899-06

**Authors:** 


ANNUAL AND ANALYTICAL CYCLOPAEDIA OF PRACTICAL MEDICINE. Volume II. By Chas. E. De M. Sajous, M. D., and one hundred associate editors, assisted by corresponding editors, collaborators and correspondents. Illustrated in black and colors. Published by the F. A. Davis Co., New York, Philadelphia and Chicago. 1899. Pages, 607. Half morocco, $5.00.

This is the second volume of a series intended to cover the entire range of medical science. The arrangement as heretofore noted, is alphabetical, and the present volume entends from "Bromide of Ethyl" to "Diphtheria." There are some mechanical advantages that should be noted.

The subject is first taken up and discussed in the usual text-book style, in large type. Then reference to particular branches of the subject not essential to a text-book discussion, but that may be of particular interest to the essayist or author, is discussed in small type; then follows the literature of the subject for 96, 97 and 98 also in small type. Thus the work is equally convenient for all classes of medical students. The mechanical execution is of the best, and for anyone wanting a current "system" of medicine it is strongly recommended.

Articles of unusual merit and current interest are Cocainomania, Diphtheria and Cerebral Hemorrhage. J.

